# A Case of a Testicular Tumor Histologically Described as a Seminoma in a Patient With an Elevated Serum Alpha-Fetoprotein

**DOI:** 10.7759/cureus.40698

**Published:** 2023-06-20

**Authors:** Kyle A Burton, Sheetal Acharya, Brian Pahn

**Affiliations:** 1 Medicine, Michigan State University College of Human Medicine, East Lansing, USA; 2 Hematology, Upper Peninsula Health Systems, Marquette, USA; 3 Pathology, Upper Peninsula Health Systems, Marquette, USA

**Keywords:** testicular seminoma, tumor markers, testicular germ cell tumors, alpha-fetoprotein, testicular cancer

## Abstract

Seminomas and non-seminomas are distinct categories of germ cell tumors arising from the testicles. This case report describes a rare occurrence of a testicular tumor exhibiting histologic characteristics of a seminoma in a 39-year-old male patient who simultaneously has an elevated serum alpha-fetoprotein (AFP). The staging characteristics of this tumor was pT1bNxM0, stage IS. This patient was treated with a right radical orchiectomy and adjuvant chemotherapy with bleomycin, etoposide, and cisplatin. This case report describes the histologic, laboratory, and imaging findings as well as the treatment and surveillance of this patient.

## Introduction

Testicular cancer is the most common cancer occurring in young males between the ages of 15 and 45 years. However, these malignancies represent only 1% of all male tumors [1]. Germ cell testicular tumors are typically categorized as either seminomas or nonseminomas. These classes of germ cell tumors may be distinguished both histologically and by the presence or absence of specific serum tumor markers. Classically, nonseminoma testicular tumors are known to secrete AFP. Seminoma testicular tumors are not known to secrete AFP, however, unspecified AFP elevations occur in approximately 2% of seminoma patients [2]. Here, we present a case of a 39-year-old non-white male who presented with a palpable right testicular nodule. After undergoing a right radical orchiectomy, further histologic examination of this tumor revealed a testicular seminoma. Pre-operative labs were significant for an elevated serum AFP, inconsistent with the diagnosis of testicular seminoma.

## Case presentation

A 39-year-old African American male with a past medical history of inguinal hernias and bilateral varicoceles presented with right testicular pain. Subsequent evaluation with scrotal ultrasound revealed a hypoechoic area in the posterior right testicular measuring 2.6 cm x 1.3 cm x 2 cm. Pre-operative tumor markers revealed normal lactate dehydrogenase (LDH) at 150 U/L, elevated beta-hCG (hCG) at 2,739 mIU/mL, and elevated AFP at 13.23 ng/mL. The patient had initially declined the recommended right radical orchiectomy. However, approximately 1 month later, he decided to proceed with surgery. The patient underwent right radical orchiectomy, and the specimen was sent to pathology. The patient was referred to Oncology for evaluation of this testicular tumor.

**Figure 1:**
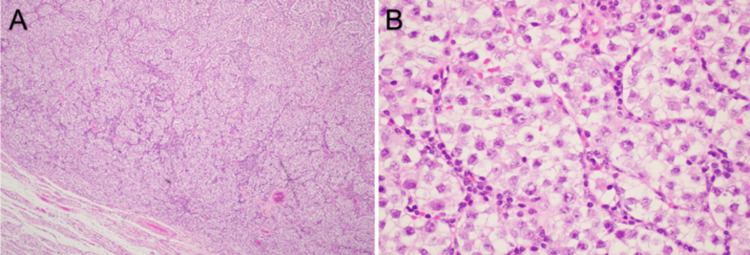
A: H&E stain of testicular tumor at A: 4X magnification and B: 40X magnification. H&E: Hematoxylin and eosin

**Figure 2:**
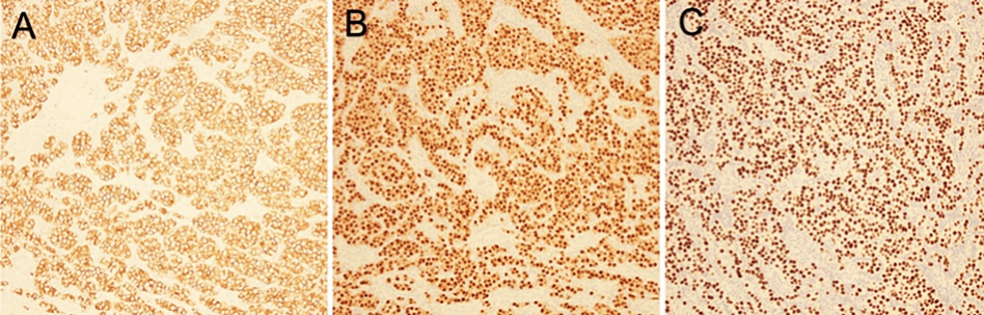
Immunohistochemical staining of testicular tumor at 10X magnification revealing positive immunohistochemical staining for A: CD117, B: OCT 3/4, and C: SALL4 CD117: Cluster of differentiation 117 OCT 3/4: Octamer binding transcription factor 3/4 SALL4: Sal-like protein 4

**Figure 3:**
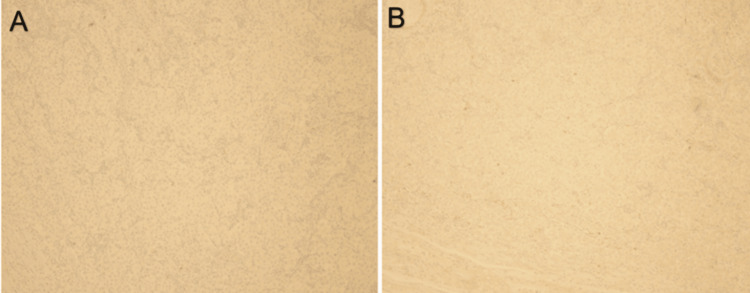
Immunohistochemical staining of testicular tumor at 10X magnification revealing negative immunohistochemical staining for A: CD30 and B: Glypican-3 CD30: Cluster of differentiation 30

Histologic evaluation showed a germ cell tumor with morphology compatible with a seminoma as shown in Figure 1. This tumor was positive for Sal-like protein 4 (SALL4), cluster of differentiation (CD) 117, and Octamer binding transcription factor (OCT) 3/4 as shown in Figure 2, and was negative for CD30 and Glypican-3 as shown in Figure 3, which further characterized this tumor as a seminoma histologically. In addition, a separate nodule of approximately 1 cm in size was identified grossly and microscopically showed a fibrous scar with associated calcifications which may have represented a burnt-out yolk sac tumor. This tumor showed the presence of germ cell neoplasia in situ and showed spread along the rete and focally in the epididymis. The tumor showed focal extension into the tunica albuginea, but no invasion of the tunica vaginalis was observed. No yolk sac component was identified morphologically or by immunohistochemical stains. The tumor was assigned the following staging characteristics: pT1bNxM0, stage IS.

Initial postoperative tumor markers were obtained approximately three weeks after surgery which revealed elevated LDH at 216 U/L, normal hCG at <0.6 IU/L, and elevated AFP at 11.9 ng/mL. Computed tomography (CT) of the chest, abdomen, and pelvis at this time was negative for metastatic disease.

Given the elevated AFP and potential burnt-out yolk sac tumor component, this tumor was determined to be a nonseminoma and was therefore treated as a nonseminoma with the following staging characteristics: pT1bNxM0, stage IS. This patient was treated with bleomycin, etoposide, and cisplatin chemotherapy and was followed with surveillance. Chemotherapy was initiated approximately two months after right radical orchiectomy. This patient’s serum AFP remained elevated despite adjuvant chemotherapy and was suspected to be due to elevated AFP at baseline. Surveillance was planned as follows: CT every 6-12 months for 2 years, then as needed. Surveillance with chest x-ray every 6 months for 2 years, then annually in years 3 and 4, and then as needed. Surveillance with serum laboratory analysis every 2 months in year 1, every 3 months in year 2, and every 6 months in years 3-5 with consideration for annual serum AFP through year 10. To date, this patient has remained without evidence of recurrent or metastatic disease.

## Discussion

Testicular tumors are most commonly discovered in young adult males. Additionally, the incidence of testicular cancer has doubled over the past 40 years [1]. Testicular malignancies are characterized by a variety of possible categories with the most common being germ-cell tumors, sex cord-gonadal stromal tumors, and secondary testicular tumors [3]. Testicular cancer often presents as an incidentally discovered palpable testicular lump [4]. The development of testicular cancer has many associated risk factors including family history of testicular cancer, cryptorchidism, exposure to diethylstilbestrol during pregnancy, and increased height [5].

Germ cell testicular tumors are typically categorized as either seminomas or nonseminomas. These germ cell tumor subtypes may be differentiated via both histologic features, and commonly with serum tumor markers including AFP, LDH, and hCG. AFP is well-known to be elevated in the setting of patients with nonseminoma germ cell tumors. Overall, germ cell testicular tumors have a 5-year survival rate of approximately 92% in children and young adults [6].

The patient in our case presented with a seminoma described histologically with an additional separate nodule which may have represented a burnt-out yolk sac tumor; however, both preoperatively and postoperatively, their serum AFP levels were elevated. Despite receiving bleomycin, etoposide, and cisplatin chemotherapy and showing no evidence of disease recurrence, this patient's serum AFP remained elevated. One case series describes the finding of serum AFP elevations in approximately 2% of patients with histologically pure seminomas arising from the testicle [2]. Another case report describes a similar case of a patient with a histologic diagnosis of a testicular seminoma who had elevated serum AFP, which was treated similarly with BEP chemotherapy [7].

This patient in our case was suspected to have an elevated serum AFP at baseline. No prior serum AFP values were available for comparison. One case report and associated literature review describes a hereditary association of elevated serum AFP, of which this patient could have a similar etiology [8]. Another paper describes patients with persistently elevated serum AFP after treatment of testicular cancer. The authors of this paper recommended a comprehensive workup for other potential etiologies of elevated serum AFP, but concluded that these patients can safely continue with surveillance if no etiologies are identified [9]. Follow-up surveillance of the patient presented in our case report included CT scans of the chest, abdomen, and pelvis, chest x-rays, and serum tumor markers, which did not reveal any evidence of recurrent or metastatic testicular malignancy.

## Conclusions

Testicular tumors histologically described as seminomas occurring simultaneously with the presence of an elevated serum AFP is a rare clinical finding as described in this case. Although rare, clinicians should be aware of the potential mismatch between histologic characterization and serum tumor marker evaluation in patients with testicular malignancies. In this case, this patient was treated with adjuvant chemotherapy including bleomycin, etoposide, and cisplatin for a histologically described seminoma with initial and persistently elevated serum AFP. The elevated postoperative serum AFP in this patient was likely due to an elevated serum AFP at baseline. This patient was determined to have favorable treatment outcomes with no evidence of recurrent disease throughout surveillance. Additional research is warranted to determine the most effective treatment options and surveillance guidelines, as well as to establish clear diagnostic criteria for the specific group of patients who may pose a diagnostic challenge.
